# Comment on “Dental Functional Status as a Possible Risk Factor of Sarcopenia: A Computed Tomography‐Based Study”

**DOI:** 10.1111/joor.13968

**Published:** 2025-04-28

**Authors:** Efsun Somay

**Affiliations:** ^1^ Faculty of Dentistry, Department of Oral and Maxillofacial Surgery Baskent University Ankara Turkey

**Keywords:** computed tomography, functional dentition, sarcopenia

Dear Editor,

We congratulate Gürhan and colleagues for their insightful study, which evaluated the sarcopenia status of participants using computerised tomography (CT) data and investigated the relationship between sarcopenia and functional dentition status [1]. The authors assessed various parameters to evaluate functional dentition in 309 patients: (1) ≥ 1 tooth in both the maxilla and mandible; (2) ≥ 10 teeth in each dental arch; (3) the presence of 12 anterior teeth; (4) 3–4 pairs of posterior occlusal premolars (POP); and (5) ≥ 1 M POP on both sides. Additionally, the authors conducted a CT‐based assessment of sarcopenia in each patient by measuring the psoas muscle area at the L3 vertebral level and Hounsfield unit radiodensity using non‐contrast images. Study results revealed that 76 (24.5%) of the patients had sarcopenia. The mean numbers of teeth were 17.12 ± 8.39 and 22.24 ± 6.72 in the sarcopenia and non‐sarcopenia groups (*p* < 0.001), respectively. Furthermore, a statistically significant inverse correlation was observed between functional dentition and sarcopenia status (*p* < 0.001). Although the study results are insightful, two concerns need to be addressed to comprehend them better.

First, according to the updated criteria of the European Working Group on Sarcopenia in the Elderly (EWGSOP‐2) [2], sarcopenia is considered probable when dynapenia (muscle strength loss) is present, and its diagnosis is confirmed when myopenia (muscle mass loss) is added to the clinical picture. EWGSOP‐2 criteria also classify the condition as severe sarcopenia when the presence of kratopenia (reduced muscle contraction capacity) is established alongside dynapenia and myopenia [2]. Thus, solely assessing muscle mass using radiological tools to measure the skeletal muscle index in cancer patients does not sufficiently fulfil the comprehensive criteria for diagnosing sarcopenia [2], as done in Gürhan and colleagues' current research [1]. Because using radiological myopenia as the sole determinant of sarcopenia may lead to exaggerating the actual rates, we recommend employing the term myopenia instead of sarcopenia to reflect the exact situation in such studies.

And second, specificity, often called the true negative rate, is a crucial metric that evaluates a test's ability to identify true negatives accurately [3, 4]. In essence, it assesses the effectiveness of a test in classifying individuals who do not possess the condition of interest. Specifically, specificity quantifies the proportion of subjects with an actual negative outcome (true negatives + false positives) who are correctly assigned a negative result, representing only the true negatives. Therefore, the 51.3% specificity rate presented in the study by Gürhan and colleagues suggests that the ability of relative tooth numbers to distinguish sarcopenia status is markedly low, indicating a significant likelihood of false results occurring in nearly half of the study population. However, upon reviewing the original Figure 2 from the manuscript and applying the well‐established Youden index [5], both the specificity and sensitivity appear to be approximately 64%, more acceptable than the estimates of 76.3% and 51.3% reported in the original Table 2. Therefore, we advise that the authors revise the receiver operating characteristic curve analysis, as this may lead to different cut‐off values and outcomes.

## Author Contributions

**Efsun Somay:** conceptualisation, investigation, methodology, project administration, validation, resources, and writing – original draft.

## Ethics Statement

The author has nothing to report.

## Conflicts of Interest

The author declares no conflicts of interest.

### Peer Review

The peer review history for this article is available at https://www.webofscience.com/api/gateway/wos/peer‐review/10.1111/joor.13968.

## Data Availability

The data produced and analysed in this study are accessible.
